# Supplementary material for: Mexican Native Black Bean Anthocyanin-Rich Extracts Modulate Biological Markers Associated with Inflammation
Source: Pharmaceuticals (Basel). 2023 Jun 13;16(6):874. doi: 10.3390/ph16060874 (PMC10301825; doi:10.3390/ph16060874)
Supplement: Supplementary file 1 [file pharmaceuticals-16-00874-s001.zip › pharmaceuticals-2426823-supplementary.pdf]

Supplementary Materials

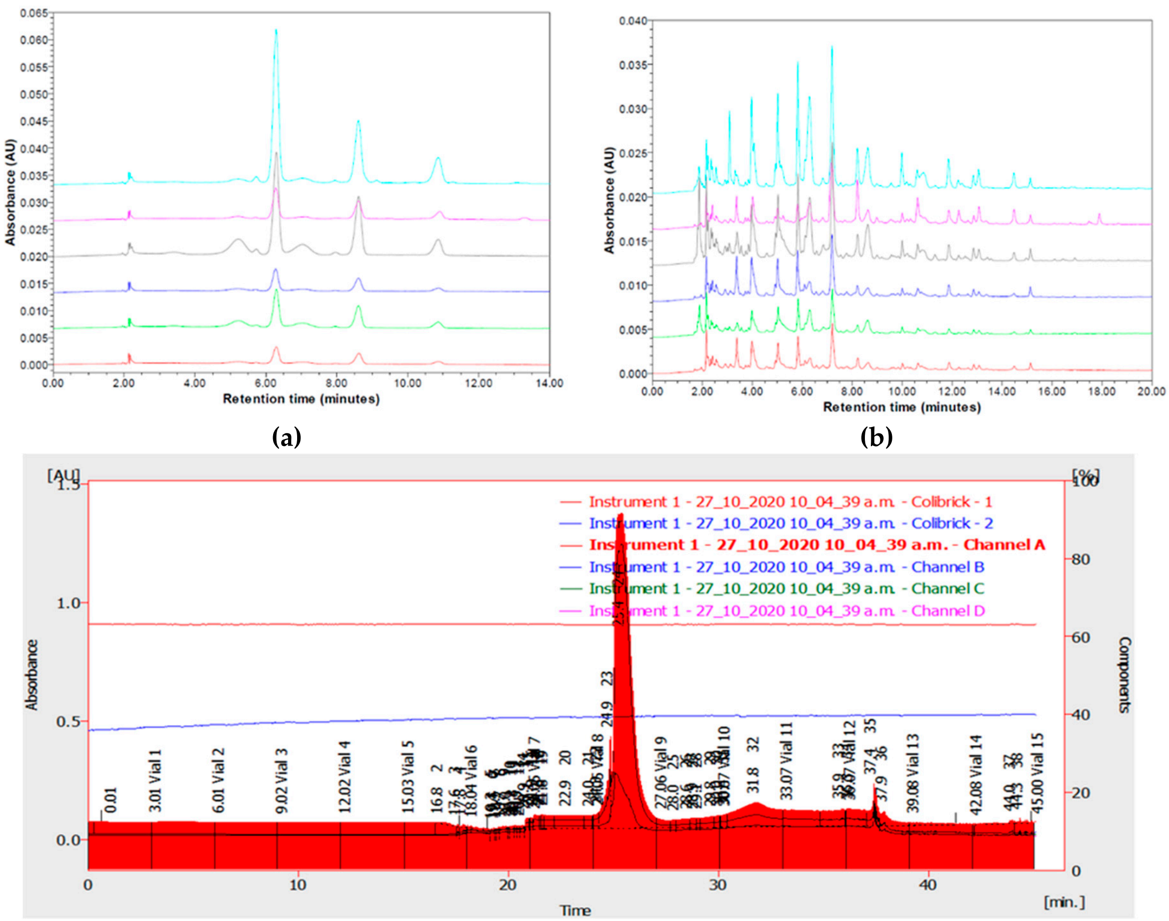

(c)

Figure S1. HPLC chromatograms by treatments. (**a**) Anthocyanins at 520 nm. (**b**) Phenolic compounds at 280 nm. (**c**) HPCCC chromatogram.
